# Crystal structure of chlorido­penta­kis(dimethyl sulfoxide-κ*O*)chromium(III) dichloride

**DOI:** 10.1107/S1600536814015852

**Published:** 2014-08-01

**Authors:** Kyung-sun Son, Jeong Oh Woo, Namhun Kim, Sung Kwon Kang

**Affiliations:** a Department of Chemistry, Chungnam National University, Daejeon 305-764, Republic of Korea

**Keywords:** crystal structure, chromium(III), dimethyl sulfoxide solvate

## Abstract

In the complex cation of the title salt, [CrCl(C_2_H_6_OS)_5_]Cl_2_, the Cr^III^ ion is coordinated by one chloride ligand and five O atoms from dimethyl sulfoxide (DMSO) ligands, leading to a slightly distorted octa­hedral coordination environment [O—Cr—O angles range from 86.69 (16) to 92.87 (16)°]. In the crystal, complex cations are arranged in hexa­gonally packed rows parallel to [010], with the chloride counter-anions situated in between. The inter­actions between cations and anions are mainly ionic in nature.

## Related literature   

For the preparation and structures of DMSO solvates of transition metal cations, see: Abbasi *et al.* (2007 ▶); Al-Najjar *et al.* (2013 ▶); Bratsos *et al.* (2013 ▶); Niu *et al.* (2012 ▶); Srivastava *et al.* (2009 ▶).
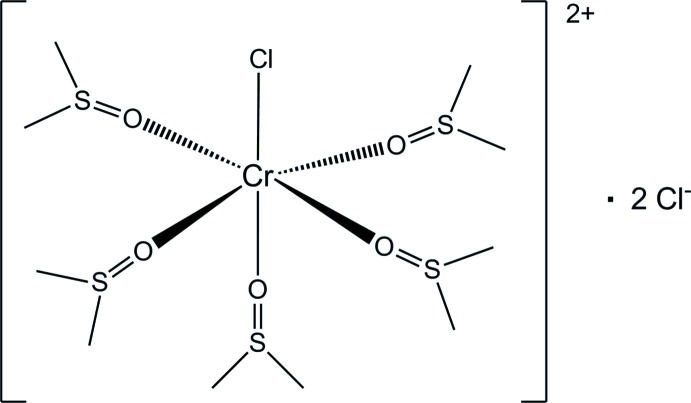


## Experimental   

### Crystal data  

[CrCl(C_2_H_6_OS)_5_]Cl_2_
*M* *_r_* = 548.99
Triclinic, 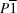

*a* = 9.5883 (4) Å
*b* = 10.4998 (4) Å
*c* = 13.0088 (5) Å
α = 70.633 (4)°
β = 83.867 (3)°
γ = 89.0009 (18)°
*V* = 1228.26 (9) Å^3^
*Z* = 2
Mo *K*α radiation
μ = 1.23 mm^−1^
*T* = 296 K
0.10 × 0.06 × 0.05 mm

### Data collection  

Bruker SMART CCD area-detector diffractometer
Absorption correction: multi-scan (*SADABS*; Bruker, 2002 ▶) *T*_min_ = 0.905, *T*_max_ = 0.935
19400 measured reflections
4576 independent reflections
2587 reflections with *I* > 2σ(*I*)
*R*_int_ = 0.115

### Refinement  

*R*[*F* ^2^ > 2σ(*F* ^2^)] = 0.072
*wR*(*F* ^2^) = 0.138
*S* = 0.98
4576 reflections
227 parameters
H-atom parameters constrained
Δρ_max_ = 0.59 e Å^−3^
Δρ_min_ = −0.47 e Å^−3^

### 

Data collection: *SMART* (Bruker, 2002 ▶); cell refinement: *SAINT* (Bruker, 2002 ▶); data reduction: *SAINT*; program(s) used to solve structure: *SHELXS2013* (Sheldrick, 2008 ▶); program(s) used to refine structure: *SHELXL2013* (Sheldrick, 2008 ▶); molecular graphics: *ORTEP-3 for Windows* (Farrugia, 2012 ▶); software used to prepare material for publication: *WinGX* (Farrugia, 2012 ▶).

## Supplementary Material

Crystal structure: contains datablock(s) global, I. DOI: 10.1107/S1600536814015852/wm5034sup1.cif

Structure factors: contains datablock(s) I. DOI: 10.1107/S1600536814015852/wm5034Isup2.hkl

**Fig. 1.**
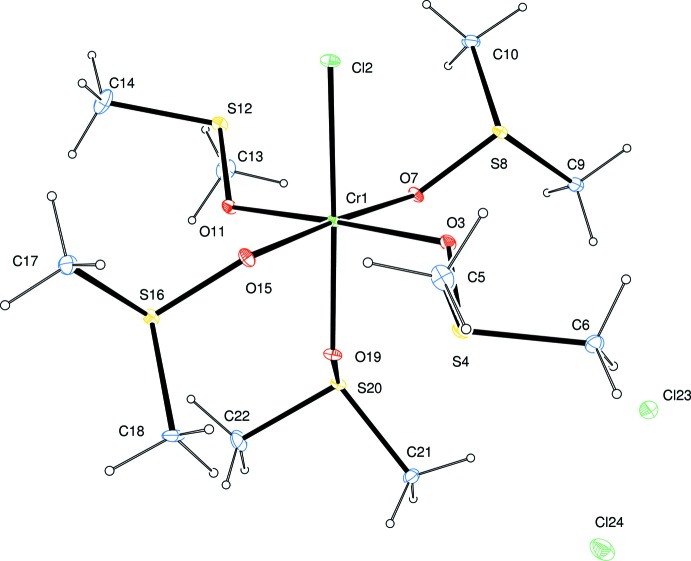
The asymmetric unit of the title compound, with the atom-numbering scheme and displacement ellipsoids drawn at the 30% probability level.

. DOI: 10.1107/S1600536814015852/wm5034fig1.tif
The asymmetric unit of the title compound, with the atom-numbering scheme and displacement ellipsoids drawn at the 30% probability level.

**Fig. 2.**
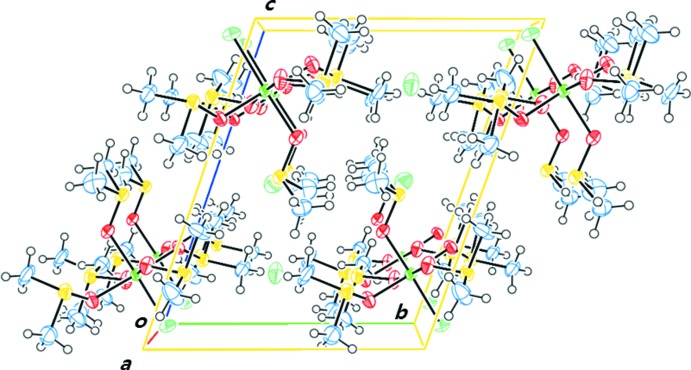
The crystal structure of the title compound in a perspective view along [100].

. DOI: 10.1107/S1600536814015852/wm5034fig2.tif
The crystal structure of the title compound in a perspective view along [100].

CCDC reference: 1012642

Additional supporting information:  crystallographic information; 3D view; checkCIF report

## Figures and Tables

**Table 1:** Selected bond lengths (Å)

Cr1—O3	1.967 (4)
Cr1—O19	1.971 (4)
Cr1—O7	1.975 (4)
Cr1—O11	1.977 (4)
Cr1—O15	1.982 (4)
Cr1—Cl2	2.3096 (18)

## supplementary crystallographic information

### S1. Experimental

All manipulations were carried out under nitrogen atmosphere using standard Schlenk techniques or in a purified nitrogen-filled drybox, and all solvents were dried and distilled prior to use according to standard methods. The title mononuclear complex was synthesized during an attempt to prepare a chromium(III) complex with 2,6-bis[(5-methyl-4-phenyl-1*H*-pyrazol-1-yl)methyl]pyridine. To a solution of CrCl_3_(THF)_3_ (0.1124 g, 0.3 mmol) in THF (6 ml) was added a solution of the organic ligand (0.126 g, 0.3 mmol) in THF (4 ml) dropwise at 333 K. The solution was stirred vigorously. A green suspension was formed immediately in 5 min, and the mixture was stirred overnight at this temperature. The product was isolated as a green powder by removing the solvent, washed repeatedly with THF followed by diethyl ether, and dried under vacuum. Bright green crystals of the title compound were obtained by slow diffusion of diethyl ether into a concentrated solution of the green solid in DMSO within one to two days.

### S2. Refinement

All H atoms were positioned geometrically and refined using a riding model, with C—H = 0.96 Å, and with *U*_iso_(H) = 1.5*U*_eq_(C).

### Crystal data

**Table:**

[CrCl(C_2_H_6_OS)_5_]Cl_2_	*Z* = 2
*M**_r_* = 548.99	*F*(000) = 570
Triclinic, *P*1	*D*_x_ = 1.484 Mg m^−^^3^
Hall symbol: -P 1	Mo *K*α radiation, λ = 0.71073 Å
*a* = 9.5883 (4) Å	Cell parameters from 2991 reflections
*b* = 10.4998 (4) Å	θ = 2.2–28.2°
*c* = 13.0088 (5) Å	µ = 1.23 mm^−^^1^
α = 70.633 (4)°	*T* = 296 K
β = 83.867 (3)°	Block, green
γ = 89.0009 (18)°	0.10 × 0.06 × 0.05 mm
*V* = 1228.26 (9) Å^3^	

### Data collection

**Table:**

Bruker SMART CCD area-detector diffractometer	2587 reflections with *I* > 2σ(*I*)
Radiation source: fine-focus sealed tube	*R*_int_ = 0.115
φ and ω scans	θ_max_ = 25.5°, θ_min_ = 1.7°
Absorption correction: multi-scan (*SADABS*; Bruker, 2002)	*h* = −11→11
*T*_min_ = 0.905, *T*_max_ = 0.935	*k* = −12→12
19400 measured reflections	*l* = −15→15
4576 independent reflections	

### Refinement

**Table:**

Refinement on *F*^2^	0 restraints
Least-squares matrix: full	Hydrogen site location: inferred from neighbouring sites
*R*[*F*^2^ > 2σ(*F*^2^)] = 0.072	H-atom parameters constrained
*wR*(*F*^2^) = 0.138	*w* = 1/[σ^2^(*F*_o_^2^) + (0.0582*P*)^2^] where *P* = (*F*_o_^2^ + 2*F*_c_^2^)/3
*S* = 0.98	(Δ/σ)_max_ < 0.001
4576 reflections	Δρ_max_ = 0.59 e Å^−^^3^
227 parameters	Δρ_min_ = −0.47 e Å^−^^3^

### Special details

**Table:**

Geometry. All e.s.d.'s (except the e.s.d. in the dihedral angle between two l.s. planes) are estimated using the full covariance matrix. The cell e.s.d.'s are taken into account individually in the estimation of e.s.d.'s in distances, angles and torsion angles; correlations between e.s.d.'s in cell parameters are only used when they are defined by crystal symmetry. An approximate (isotropic) treatment of cell e.s.d.'s is used for estimating e.s.d.'s involving l.s. planes.

### Fractional atomic coordinates and isotropic or equivalent isotropic displacement parameters (Å^2^)

**Table:**

	*x*	*y*	*z*	*U*_iso_*/*U*_eq_	
Cr1	0.73481 (10)	0.87030 (9)	0.21633 (7)	0.0237 (3)	
Cl2	0.75680 (18)	1.04916 (16)	0.05360 (12)	0.0396 (4)	
O3	0.5471 (4)	0.8278 (4)	0.1872 (3)	0.0355 (11)	
S4	0.49654 (17)	0.68654 (16)	0.19631 (13)	0.0332 (4)	
C5	0.5051 (8)	0.6874 (7)	0.0590 (5)	0.055 (2)	
H5A	0.4633	0.768	0.0149	0.082*	
H5B	0.4554	0.6098	0.0571	0.082*	
H5C	0.6014	0.6849	0.0307	0.082*	
C6	0.3132 (6)	0.6983 (7)	0.2227 (5)	0.0436 (18)	
H6A	0.291	0.7047	0.2946	0.065*	
H6B	0.268	0.6194	0.2189	0.065*	
H6C	0.281	0.7771	0.1691	0.065*	
O7	0.6490 (4)	0.9863 (4)	0.2973 (3)	0.0296 (10)	
S8	0.53284 (17)	1.08678 (15)	0.25183 (13)	0.0306 (4)	
C9	0.4206 (7)	1.0783 (6)	0.3701 (5)	0.0445 (18)	
H9A	0.3776	0.9901	0.4009	0.067*	
H9B	0.3492	1.1448	0.3511	0.067*	
H9C	0.4737	1.0956	0.4228	0.067*	
C10	0.6119 (7)	1.2474 (6)	0.2266 (5)	0.0452 (19)	
H10A	0.6571	1.2467	0.2892	0.068*	
H10B	0.5413	1.3151	0.2133	0.068*	
H10C	0.6803	1.2672	0.1635	0.068*	
O11	0.9199 (4)	0.9089 (4)	0.2546 (3)	0.0327 (11)	
S12	0.97576 (18)	1.05332 (16)	0.23286 (13)	0.0342 (4)	
C13	1.0000 (8)	1.0568 (8)	0.3636 (5)	0.057 (2)	
H13A	0.9107	1.0637	0.4022	0.086*	
H13B	1.0583	1.1332	0.3567	0.086*	
H13C	1.0444	0.9753	0.4034	0.086*	
C14	1.1538 (7)	1.0469 (8)	0.1831 (6)	0.067 (3)	
H14A	1.1992	0.9765	0.2358	0.101*	
H14B	1.1995	1.1319	0.1713	0.101*	
H14C	1.1593	1.0286	0.1152	0.101*	
O15	0.8145 (4)	0.7458 (4)	0.1404 (3)	0.0339 (11)	
S16	0.93855 (17)	0.65183 (16)	0.17455 (13)	0.0339 (4)	
C17	1.0204 (7)	0.6520 (7)	0.0464 (5)	0.053 (2)	
H17A	0.9518	0.6318	0.0051	0.079*	
H17B	1.0917	0.5849	0.0575	0.079*	
H17C	1.0621	0.7393	0.0068	0.079*	
C18	0.8644 (8)	0.4878 (6)	0.2216 (6)	0.061 (2)	
H18A	0.8188	0.469	0.2946	0.091*	
H18B	0.937	0.4235	0.2219	0.091*	
H18C	0.797	0.4812	0.174	0.091*	
O19	0.7205 (4)	0.7130 (4)	0.3520 (3)	0.0343 (11)	
S20	0.73602 (17)	0.72155 (15)	0.46602 (13)	0.0306 (4)	
C21	0.6037 (6)	0.6113 (6)	0.5523 (5)	0.0422 (18)	
H21A	0.5134	0.6452	0.5318	0.063*	
H21B	0.6103	0.6051	0.6269	0.063*	
H21C	0.615	0.5234	0.5453	0.063*	
C22	0.8822 (7)	0.6230 (8)	0.5089 (6)	0.062 (2)	
H22A	0.874	0.5383	0.4966	0.092*	
H22B	0.8864	0.6068	0.5856	0.092*	
H22C	0.9661	0.6698	0.4681	0.092*	
Cl23	0.25112 (19)	0.74394 (19)	0.49760 (15)	0.0542 (5)	
Cl24	0.2423 (2)	0.37459 (18)	0.19086 (17)	0.0579 (6)	

### Atomic displacement parameters (Å^2^)

**Table:**

	*U*^11^	*U*^22^	*U*^33^	*U*^12^	*U*^13^	*U*^23^
Cr1	0.0290 (6)	0.0197 (5)	0.0227 (5)	0.0021 (4)	−0.0077 (4)	−0.0062 (4)
Cl2	0.0555 (12)	0.0340 (10)	0.0231 (9)	0.0017 (8)	−0.0081 (8)	0.0000 (7)
O3	0.034 (3)	0.023 (2)	0.053 (3)	0.002 (2)	−0.012 (2)	−0.014 (2)
S4	0.0379 (11)	0.0255 (9)	0.0383 (10)	0.0026 (8)	−0.0171 (8)	−0.0094 (8)
C5	0.063 (5)	0.066 (5)	0.049 (5)	0.014 (4)	−0.016 (4)	−0.034 (4)
C6	0.041 (4)	0.038 (4)	0.051 (5)	−0.008 (3)	−0.008 (4)	−0.013 (4)
O7	0.039 (3)	0.029 (2)	0.027 (2)	0.013 (2)	−0.012 (2)	−0.015 (2)
S8	0.0377 (10)	0.0240 (9)	0.0313 (10)	0.0053 (8)	−0.0101 (8)	−0.0091 (8)
C9	0.047 (5)	0.035 (4)	0.044 (4)	0.008 (4)	0.000 (4)	−0.005 (3)
C10	0.056 (5)	0.029 (4)	0.043 (4)	0.002 (4)	−0.002 (4)	−0.003 (3)
O11	0.029 (2)	0.026 (2)	0.045 (3)	−0.001 (2)	−0.018 (2)	−0.010 (2)
S12	0.0416 (11)	0.0294 (10)	0.0357 (10)	0.0013 (8)	−0.0127 (8)	−0.0137 (8)
C13	0.067 (5)	0.078 (6)	0.039 (5)	−0.003 (4)	−0.001 (4)	−0.037 (4)
C14	0.056 (5)	0.081 (6)	0.079 (6)	−0.026 (5)	0.026 (5)	−0.054 (5)
O15	0.038 (3)	0.033 (3)	0.039 (3)	0.011 (2)	−0.015 (2)	−0.021 (2)
S16	0.0357 (10)	0.0292 (10)	0.0389 (10)	0.0055 (8)	−0.0094 (8)	−0.0124 (8)
C17	0.053 (5)	0.043 (5)	0.057 (5)	0.003 (4)	0.011 (4)	−0.014 (4)
C18	0.073 (6)	0.023 (4)	0.064 (5)	−0.001 (4)	0.014 (4)	0.009 (4)
O19	0.050 (3)	0.021 (2)	0.028 (3)	0.002 (2)	−0.007 (2)	−0.0026 (19)
S20	0.0389 (10)	0.0239 (9)	0.0280 (9)	0.0043 (8)	−0.0089 (8)	−0.0057 (7)
C21	0.039 (4)	0.044 (4)	0.037 (4)	−0.007 (3)	0.002 (3)	−0.007 (3)
C22	0.041 (5)	0.074 (6)	0.067 (6)	0.020 (4)	−0.027 (4)	−0.014 (5)
Cl23	0.0512 (12)	0.0524 (12)	0.0527 (12)	0.0007 (10)	0.0000 (10)	−0.0108 (10)
Cl24	0.0641 (13)	0.0360 (11)	0.0797 (15)	0.0096 (9)	−0.0272 (11)	−0.0217 (10)

### Geometric parameters (Å, º)

**Table:**

Cr1—O3	1.967 (4)	S12—C14	1.768 (7)
Cr1—O19	1.971 (4)	C13—H13A	0.96
Cr1—O7	1.975 (4)	C13—H13B	0.96
Cr1—O11	1.977 (4)	C13—H13C	0.96
Cr1—O15	1.982 (4)	C14—H14A	0.96
Cr1—Cl2	2.3096 (18)	C14—H14B	0.96
O3—S4	1.531 (4)	C14—H14C	0.96
S4—C6	1.764 (6)	O15—S16	1.539 (4)
S4—C5	1.777 (6)	S16—C18	1.760 (6)
C5—H5A	0.96	S16—C17	1.765 (6)
C5—H5B	0.96	C17—H17A	0.96
C5—H5C	0.96	C17—H17B	0.96
C6—H6A	0.96	C17—H17C	0.96
C6—H6B	0.96	C18—H18A	0.96
C6—H6C	0.96	C18—H18B	0.96
O7—S8	1.546 (4)	C18—H18C	0.96
S8—C9	1.758 (6)	O19—S20	1.538 (4)
S8—C10	1.775 (6)	S20—C22	1.755 (6)
C9—H9A	0.96	S20—C21	1.760 (6)
C9—H9B	0.96	C21—H21A	0.96
C9—H9C	0.96	C21—H21B	0.96
C10—H10A	0.96	C21—H21C	0.96
C10—H10B	0.96	C22—H22A	0.96
C10—H10C	0.96	C22—H22B	0.96
O11—S12	1.542 (4)	C22—H22C	0.96
S12—C13	1.753 (6)		
			
O3—Cr1—O19	90.28 (17)	O11—S12—C13	103.8 (3)
O3—Cr1—O7	89.61 (16)	O11—S12—C14	103.4 (3)
O19—Cr1—O7	90.78 (16)	C13—S12—C14	98.5 (4)
O3—Cr1—O11	176.76 (18)	S12—C13—H13A	109.5
O19—Cr1—O11	86.97 (17)	S12—C13—H13B	109.5
O7—Cr1—O11	88.70 (16)	H13A—C13—H13B	109.5
O3—Cr1—O15	88.69 (16)	S12—C13—H13C	109.5
O19—Cr1—O15	86.69 (16)	H13A—C13—H13C	109.5
O7—Cr1—O15	176.95 (18)	H13B—C13—H13C	109.5
O11—Cr1—O15	92.87 (16)	S12—C14—H14A	109.5
O3—Cr1—Cl2	90.00 (13)	S12—C14—H14B	109.5
O19—Cr1—Cl2	177.61 (13)	H14A—C14—H14B	109.5
O7—Cr1—Cl2	91.59 (12)	S12—C14—H14C	109.5
O11—Cr1—Cl2	92.82 (13)	H14A—C14—H14C	109.5
O15—Cr1—Cl2	90.95 (13)	H14B—C14—H14C	109.5
S4—O3—Cr1	124.5 (2)	S16—O15—Cr1	125.3 (2)
O3—S4—C6	101.8 (3)	O15—S16—C18	104.8 (3)
O3—S4—C5	105.0 (3)	O15—S16—C17	101.7 (3)
C6—S4—C5	99.0 (3)	C18—S16—C17	98.8 (3)
S4—C5—H5A	109.5	S16—C17—H17A	109.5
S4—C5—H5B	109.5	S16—C17—H17B	109.5
H5A—C5—H5B	109.5	H17A—C17—H17B	109.5
S4—C5—H5C	109.5	S16—C17—H17C	109.5
H5A—C5—H5C	109.5	H17A—C17—H17C	109.5
H5B—C5—H5C	109.5	H17B—C17—H17C	109.5
S4—C6—H6A	109.5	S16—C18—H18A	109.5
S4—C6—H6B	109.5	S16—C18—H18B	109.5
H6A—C6—H6B	109.5	H18A—C18—H18B	109.5
S4—C6—H6C	109.5	S16—C18—H18C	109.5
H6A—C6—H6C	109.5	H18A—C18—H18C	109.5
H6B—C6—H6C	109.5	H18B—C18—H18C	109.5
S8—O7—Cr1	121.5 (2)	S20—O19—Cr1	123.9 (2)
O7—S8—C9	103.2 (3)	O19—S20—C22	104.6 (3)
O7—S8—C10	103.8 (3)	O19—S20—C21	103.8 (3)
C9—S8—C10	98.7 (3)	C22—S20—C21	98.7 (3)
S8—C9—H9A	109.5	S20—C21—H21A	109.5
S8—C9—H9B	109.5	S20—C21—H21B	109.5
H9A—C9—H9B	109.5	H21A—C21—H21B	109.5
S8—C9—H9C	109.5	S20—C21—H21C	109.5
H9A—C9—H9C	109.5	H21A—C21—H21C	109.5
H9B—C9—H9C	109.5	H21B—C21—H21C	109.5
S8—C10—H10A	109.5	S20—C22—H22A	109.5
S8—C10—H10B	109.5	S20—C22—H22B	109.5
H10A—C10—H10B	109.5	H22A—C22—H22B	109.5
S8—C10—H10C	109.5	S20—C22—H22C	109.5
H10A—C10—H10C	109.5	H22A—C22—H22C	109.5
H10B—C10—H10C	109.5	H22B—C22—H22C	109.5
S12—O11—Cr1	123.0 (2)		
