# Supplementary figures and images for: Crystal structure of chlorido­penta­kis(dimethyl sulfoxide-κO)chromium(III) dichloride
Source: Acta Crystallogr Sect E Struct Rep Online. 2014 Aug 1;70(Pt 9):m309. doi: 10.1107/S1600536814015852 (PMC4186071; doi:10.1107/S1600536814015852)

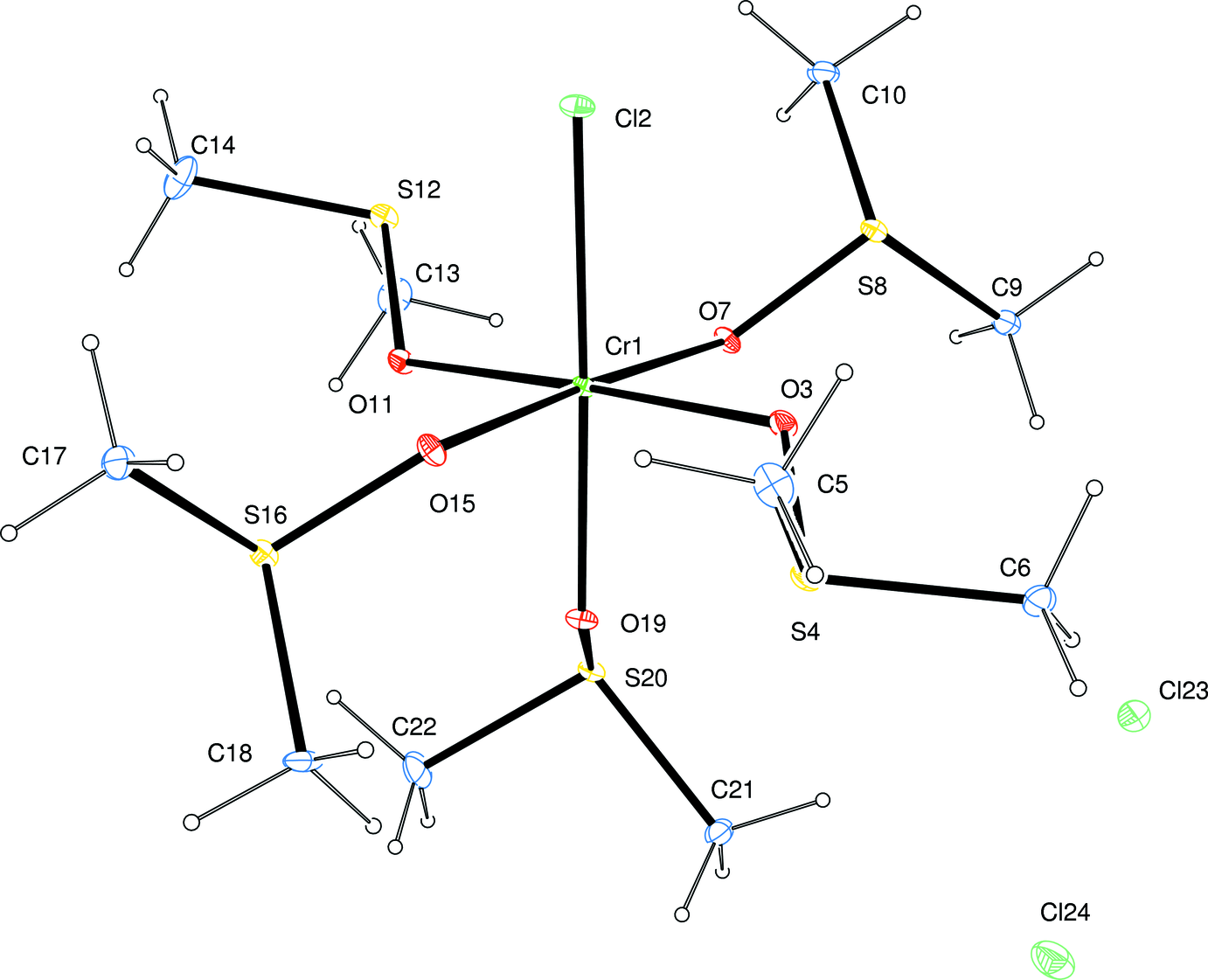

Supplement: Supplementary file 3 [file e-70-0m309-fig1.tif]

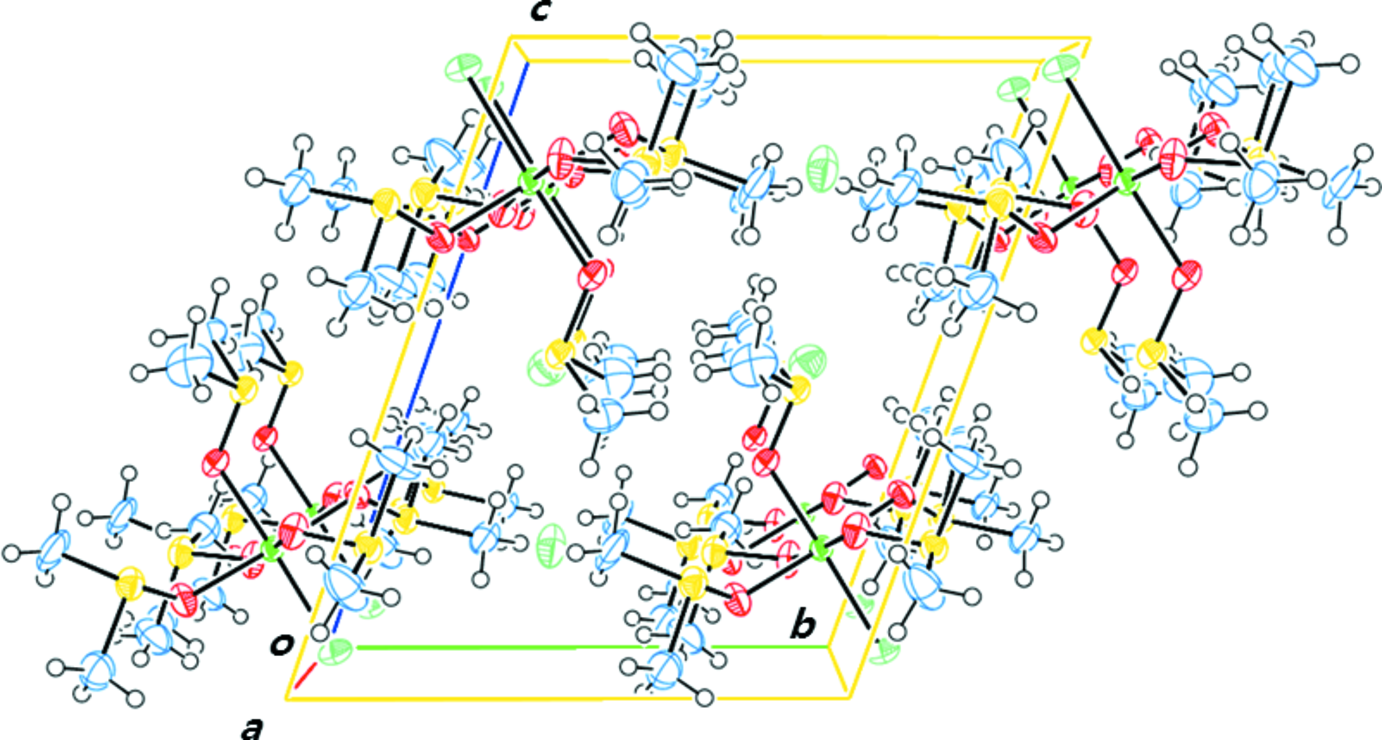

Supplement: Supplementary file 4 [file e-70-0m309-fig2.tif]
